# Phenotypic and Genetic Analyses of Mastitis, Endometritis, and Ketosis on Milk Production and Reproduction Traits in Chinese Holstein Cattle

**DOI:** 10.3390/ani14162372

**Published:** 2024-08-15

**Authors:** Xiaoli Ren, Haibo Lu, Yachun Wang, Lei Yan, Changlei Liu, Chu Chu, Zhuo Yang, Xiangnan Bao, Mei Yu, Zhen Zhang, Shujun Zhang

**Affiliations:** 1 Frontiers Science Center for Animal Breeding and Sustainable Production, Huazhong Agricultural University, Wuhan 430070, China; renxiaoli@webmail.hzau.edu.cn (X.R.); chu1999@webmail.hzau.edu.cn (C.C.); yang_zhuo@webmail.hzau.edu.cn (Z.Y.); 18548163596@163.com (X.B.); yumei@mail.hzau.edu.cn (M.Y.); 2 Key Lab of Agricultural Animal Genetics, Breeding and Reproduction of Ministry of Education, Huazhong Agricultural University, Wuhan 430070, China; 3 Henan Dairy Herd Improvement Center, Zhengzhou 450046, China; 4 College of Animal Science and Technology, China Agricultural University, Beijing 100193, China; luhaibo979@163.com (H.L.); wangyachun@cau.edu.cn (Y.W.); 5 Henan Seed Industry Development Center, Zhengzhou 450046, China; yanleihcy@163.com; 6 Henan Dairy Herd Improvement Co., Ltd., Zhengzhou 450046, China; c@hndhi.com

**Keywords:** mastitis, endometritis, ketosis, milk production traits, reproduction traits, genetic analysis, Chinese Holstein Cattle

## Abstract

**Simple Summary:**

The aims of this study are to analyze prevalence rates, influence, and genetic parameters (heritability, genetic, and phenotypic correlations) of mastitis (MAS), endometritis (MET), and ketosis (KET) in Chinese Holstein Cattle. Data from 37,836 dairy cows in Central China were analyzed using the logistic regression method, a mixed linear model, and an animal model (BLUP). The prevalence rates of MAS, MET, and KET were 20.04%, 10.68%, and 7.33%, respectively. MAS and MET had a negative effect on production traits. High-milk yield cows had high KET prevalence rates. MAS, MET, and KET all had negative influences on reproduction traits. The heritabilities of predispositions to MAS, MET, and KET were 0.09, 0.01, and 0.02, respectively.

**Abstract:**

Mastitis (MAS), endometritis (MET), and ketosis (KET) are prevalent diseases in dairy cows that result in substantial economic losses for the dairy farming industry. This study gathered 26,014 records of the health and sickness of dairy cows and 99,102 data of reproduction from 13 Holstein dairy farms in Central China; the milk protein and milk fat content from 56,640 milk samples, as well as the pedigree data of 37,836 dairy cows were obtained. The logistic regression method was used to analyze the variations in the prevalence rates of MAS, MET, and KET among various parities; the mixed linear model was used to examine the effects of the three diseases on milk production, milk quality, and reproductive traits. DMU software (version 5.2) utilized the DMUAI module in conjunction with the single-trait and two-trait animal model, as well as best linear unbiased prediction (BLUP), to estimate the genetic parameters for the three diseases, milk production, milk quality, and reproductive traits in dairy cows. The primary findings of the investigation comprised the following: (1) The prevalence rates of MAS, MET, and KET in dairy farms were 20.04%, 10.68%, and 7.33%, respectively. (2) MAS and MET had a substantial impact (*p <* 0.01) on milk production, resulting in significant decreases of 112 kg and 372 kg in 305-d Milk Yield (305-d MY), 4 kg and 12 kg in 305-d Protein Yield (305-d PY), and 6 kg and 16 kg in 305-d Fat Yield (305-d FY). As a result of their excessive 305-d MY, some cows were diagnosed with KET due to glucose metabolism disorder. The 305-d MY of cows with KET was significantly higher than that of healthy cows (205 kg, *p <* 0.01). (3) All three diseases resulted in an increase in the Interval from Calving to First Service (CTFS, 0.60–1.50 d), Interval from First Service to Conception (FSTC, 0.20–16.20 d), Calving Interval (CI, 4.00–7.00 d), and Number of Services (NUMS, 0.07–0.35). (4) The heritabilities of cows with MAS, MET, and KET were found to be low, with values of 0.09, 0.01, and 0.02, respectively. The genetic correlation between these traits ranged from 0.14 to 0.44. This study offers valuable insights on the prevention and control of the three diseases, as well as feeding management and genetic breeding.

## 1. Introduction

Dairy cow MAS, MET, and KET are prevalent diseases in large-scale and intensive feeding farms. It was reported that the MAS prevalence rate ranges from 0.60% to 18.2% [1], the MET prevalence rate ranges from 3.4% to 53% [2,3], and the KET prevalence rate is from 10% to 30% [4]. These diseases result in increased expenses for veterinary testing and treatment, labor costs, and the culling rate of dairy cows, therefore impacting production performance, reproduction efficiency, and the economic profitability of the farm [5,6,7,8,9]. Studies have shown that MAS results in economic losses ranging from USD 325.76 to USD 444.00 per case and EUR 70.65 to EUR 365.00 per case [10,11]. KET is associated with economic losses ranging from USD 77.00 to USD 203.00 US per case and EUR 150.41 to EUR 259.00 per case [12]. Additionally, MET leads to economic losses ranging from USD 171.69 to USD 262.65 per case [11,13].

The prioritization of a high milk yield in breeding, without considering the selection of health and reproductive traits, has deteriorated dairy cows’ health and reproductive traits [11]. Genetic improvement effectively reduces MAS, MET, and KET prevalence rates in dairy cows by selectively breeding cows that are not prone to illnesses [14,15]. The heritabilities of cows with MAS, MET, and KET range from 0.01 to 0.12, 0.02 to 0.03, and 0.03 to 0.16, respectively [11,16,17,18,19,20]. Furthermore, MAS, MET, and KET have previously been included in the Total Performance Index and the Net Merit Index in the United States [21,22].

There are differences in disease diagnosis methods across various nations, regions, and dairy farms, resulting in disparities in the prevalence rates of the three diseases. Additionally, the genetic parameters of cows with the diseases also have population specificity. Studies and publications on the prevalence rates and genetic parameters of dairy cow MAS, MET, and KET are scarce in Central China, the main milk-producing region in China. Furthermore, the effects of these three diseases on dairy cow milk production, milk quality, and reproductive traits in Central China remain uncertain. This study involved collecting data on the health and illness of dairy cows and the production, reproduction, and pedigree of cows from 13 dairy farms in Central China. Our analyses focused on three main areas: (1) examining the prevalence rates and changes among different parities of three diseases; (2) investigating the effects of these diseases on milk production, milk quality, and reproductive traits; and (3) determining the genetic parameters of these diseases, milk production, milk quality, and reproductive traits. This study aims to establish a basis for comprehending the prevalence rates of MAS, MET, and KET and their impacts on the essential traits of dairy cows in Central China. Additionally, it seeks to contribute to the effective management of and breeding strategies for these three diseases.

## 2. Materials and Methods

### 2.1. Determination, Collection, Complication, and Analyses of Data on Dairy Cow Traits

Data on diseases such as MAS, MET, and KET in dairy cows and reproductive data, including calving, mating, and pregnancy, were collected from 13 dairy farms in Central China. Monthly milk yield data and milk samples were collected from lactating cows, and the protein percentage, fat percentage, somatic cell count (SCC), and differential somatic cell count (DSCC) were measured. Finally, 14 traits in four types (diseases, milk quality, milk production, and reproduction) were presented in Table 1. Dairy cows were reared using a total mixed ration (TMR) and provided with drinking water through an automated drinking fountain in the cowshed, utilizing advanced feeding management techniques. The dairy farm was in a warm-temperate, subtropical region with a humid to semi-humid monsoon climate. The data records for the farm were relatively comprehensive.

Disease data of MAS, MET, and KET: A total of 26,014 disease records were gathered from 19,587 dairy cows. Specifically, there were 26,683 recordings of MAS, 8936 reports of MET, and 6337 records of KET. All clinical disease data were recorded in the dairy farm data management system by veterinarians. The detection methods included the California Mastitis Test for mastitis in milking cows, observing the purulent state of vaginal secretions for endometritis in fresh cows, and measuring blood β-hydroxybutyrate using a handheld meter for ketosis in fresh cows.

Milk quality data: A total of 855,540 data, comprising protein percentage, fat percentage, and SCC, were obtained by collecting and analyzing milk samples in the DHI laboratory in Zhengzhou, Henan. The SCC was transformed into a somatic cell score (SCS) [23]. The average SCS in lactation (ASCS) was determined for each dairy cow during each lactation period, along with the average DSCC in lactation (ADSCC), Average Protein Percentage in lactation (APROPER), and Average Fat Percentage in lactation (AFATPER). A total of 56,640 milk quality data were collected from 34,884 dairy cows.

Milk production data of 305-d MY, 305-d FY, and 305-d PY: The nls function and the Wood function [24] in the stats package of the R software (version 4.3.2) [25] were employed to fit the lactation curves of dairy cows in 1 to 5 parities. The parameter values of a, b, and c of the Wood function were calculated, along with three milk production traits: 305-d MY, 305-d FY, and 305-d PY. A total of 42,665 data information were calculated from 28,522 dairy cows.

Reproductive traits data: Data were acquired from 11 dairy farms with comprehensive reproductive records, including information on the calving, mating, first inspection, and re-inspection of dairy cows. The CTFS, FSTC, NUMS, and CI were calculated. Ultimately, a total of 99,102 reproductive records during lactation were gathered from 44,641 dairy cows.

Additionally, a pedigree analysis was conducted for dairy cows, and animals lacking information on either their sire, dam, or both parents were excluded. Finally, a total of 70,833 data containing complete pedigree records and four types of traits were obtained from 37,836 dairy cows. The detailed pedigree information was as follows: (1) 37,836 dairy cows with a parental generation pedigree, including 1499 sires and 30,161 dams; (2) 32,966 dairy cows with a grandparental generation pedigree, including 1872 sires and 17,253 dams; (3) 32,861 dairy cows with a great-grandparental generation pedigree, including 1484 sires and 8165 dams.

### 2.2. Statistical Analysis Method and Software

Analyses of the prevalence rates of MAS, MET, and KET: The logistic regression method of the glm function in the stats package of the R software [25] was used to analyze the changes in the prevalence rates of MAS, MET, and KET in different parities. The logistic regression model was as follows:

(1) $$logit(p)=\ln\left(\frac{p}{1-p}\right)=\alpha +\beta_{1}x_{1}$$

where *p* represents the probability of the occurrence of MAS, MET, or KET. p represents the probability of the occurrence of an event, specifically the chance of illness; 1−p represents the probability of the non-occurrence of the event. α represents a constant value, *β*_1_ represents the variable coefficient, and $x_{1}$ represents the parity.

The odds ratio (OR) and *p*-value were calculated by logistic regression using the following formula:

(2) $$p=\frac{exp(\alpha +\beta_{1}x_{1})}{1+exp(\alpha +\beta_{1}x_{1})}$$

(3) $$OR=\frac{p}{1+p}$$

where p represents the probability of the occurrence of an event, specifically the chance of illness. The *OR* value represents the effect on the dependent variable when the independent variable rises by one unit.

The influence of the three diseases on milk production, milk quality, and reproductive trait: The R software [25] lme4 package [26] was utilized to analyze the impacts of MAS, MET, and KET on milk production, milk quality, and reproductive traits. Additionally, the lsmeans package [27] was used to perform multiple comparisons to examine the relationship between disease status (0 indicating not being ill and 1 indicating being ill) and milk production, milk quality, and reproductive trait. The analytical model is outlined as follows:

(4) $$y_{ijklmnopqr}=H_{i}+{Par}_{j}+{BS}_{k}+{CS}_{l}+{Ageofm}_{m}+{MAS}_{n}+{KET}_{o}+{MET}_{p}+a_{q}+e_{ijklmnopqr}$$

where $y_{ijklmnopqr}$ represents the phenotypic data of the milk production, milk quality or reproductive traits of cows *q*; $H_{i}$ represents the impact of the dairy farm; ${Par}_{j}$ represents the parity effect; ${BS}_{k}$ represents the impact of the birth season–year; ${CS}_{l}$ represents the calving season–year effect; ${Ageofm}_{m}$ represents the random effect of the calving month; ${MAS}_{n}$ represents the presence of MAS in the dairy cow at this parity;${KET}_{o}$ represents the presence of KET in the dairy cow during this particular parity; ${MET}_{p}$ represents the presence of MET in the dairy cow at this particular parity; $a_{q}$ represents the individual random effect; and $e_{ijklmnopqr}$ represents the residual. The grouping of the seasons was as follows: (1) first season: March, April, and May; (2) second season: June, July, and August; (3) third season: September, October, and November; and (4) fourth season: December, January, and February.

Genetic analyses of the three diseases, milk production, milk quality, and reproductive trait: The DMUAI module of the DMU software [28] was used in conjunction with the BLUP method of the single trait animal model to calculate the genetic parameters for traits in dairy cows. The study used a two-trait animal model BLUP to calculate the genetic and phenotypic correlations between these traits. The model is as follows:

(5) $$y_{ijklmno}=H_{i}+{Par}_{j}+{BS}_{k}+{CS}_{l}+{Ageofm}_{m}+a_{n}+e_{ijklmno}$$

where $y_{ijklmno}$ represents diseases, milk production, milk quality, or reproductive trait;$H_{i}$ represents the effect of the dairy farm; ${Par}_{j}\;$ represents the parity effect; ${BS}_{k}$ represents the effect of the birth season–year;${CS}_{l}$ represents the calving season–year effect; ${Ageofm}_{m}$ represents the random effect of the age of calving; $a_{q}$ represents the individual random effect; and $e_{ijklmnopqr}$ represents the residual.

The formulas for heritability, genetic correlation, phenotypic correlation, and the standard error of heritability [29] are as follows:

(6) $$h^{2}=\frac{\sigma_{a}^{2}}{\sigma_{a}^{2}+\sigma_{e}^{2}}$$

(7) $$r_{A}=\frac{Cov(a_{1},a_{2})}{\sqrt{\sigma_{a_{1}}^{2}\sigma_{a_{2}}^{2}}}$$

(8) $$r_{P}=\frac{Cov(p_{1},p_{2})}{\sqrt{\sigma_{p_{1}}^{2}\sigma_{p_{2}}^{2}}}$$

(9) $${SE}^{2}={\left[\frac{\sigma_{a}^{2}}{\sigma_{p}^{2}}\right]}^{2}\left[\frac{Var\left(\sigma_{a}^{2}\right)}{{\left(\sigma_{a}^{2}\right)}^{2}}+\frac{Var\left(\sigma_{p}^{2}\right)}{{\left(\sigma_{p}^{2}\right)}^{2}}-2\times \frac{Cov\left(\sigma_{a}^{2}{,\sigma }_{p}^{2}\right)}{\sigma_{a}^{2}\sigma_{p}^{2}}\right]$$

where $h^{2}$: heritability; $\sigma_{a}^{2}$: additive genetic variance; $\sigma_{e}^{2}$: residual variance; $r_{A}$: genetic correlation between any two traits represents the correlation between additive genetic effects; $r_{P}$: phenotypic correlation between traits;$Cov\left(a_{1},a_{2}\right)$: the covariance of the additive effects of traits $a_{1}$ and $a_{2}$; and $Cov\left(p_{1},p_{2}\right)$: phenotypic covariance of traits $p_{1}$ and $p_{2}$. A *t*-test was used for the testing of heritability, genetic correlation, and phenotypic correlation.

## 3. Results

### 3.1. Prevalence Rates of MAS, MET, and KET and Their Change Characteristics with Parity

The prevalence rate of MAS was 20.04 (7.75)%, with a range of 8.85% to 29.43% seen in different dairy farms. The overall prevalence rate of MET was 10.68 (6.44)%, with prevalence rates at different dairy farms that ranged from 2.47% to 21.28%. The overall prevalence rate of KET was 7.33 (6.57)%, with prevalence rates ranging from 0.75% to 25.35% at different dairy farms.

**Figure 1:**
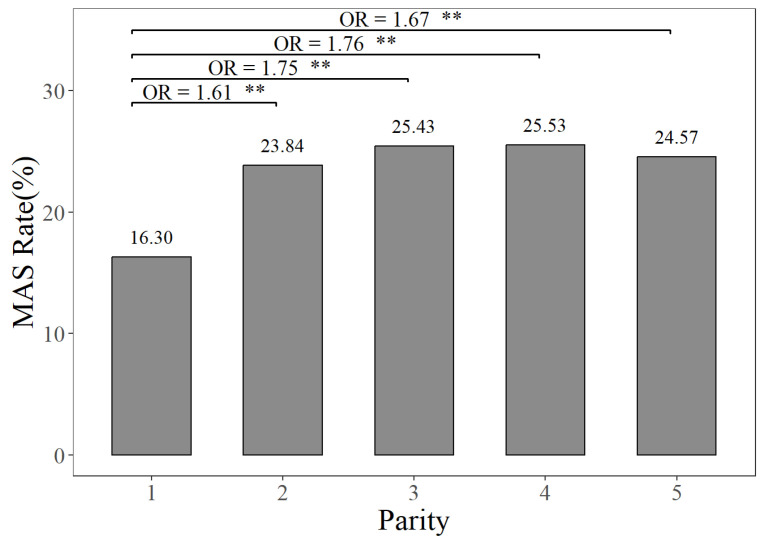
The prevalence rates and odds ratios (ORs) of mastitis in different parities, compared to the first parity. The MAS Rate represents the mastitis prevalence rate. “**” represents no and highly significant differences (*p* < 0.01).

**Figure 2:**
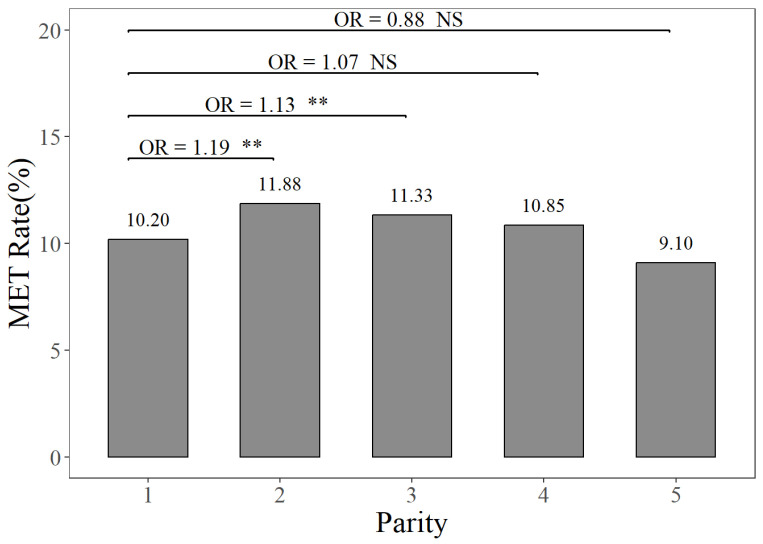
The prevalence rates and odds ratios (ORs) of endometritis in different parities, compared to the first parity. The MET Rate represents the endometritis prevalence rate. NS and “**”, respectively, represent no and highly significant differences (*p >* 0.05, *p <* 0.01).

**Figure 3:**
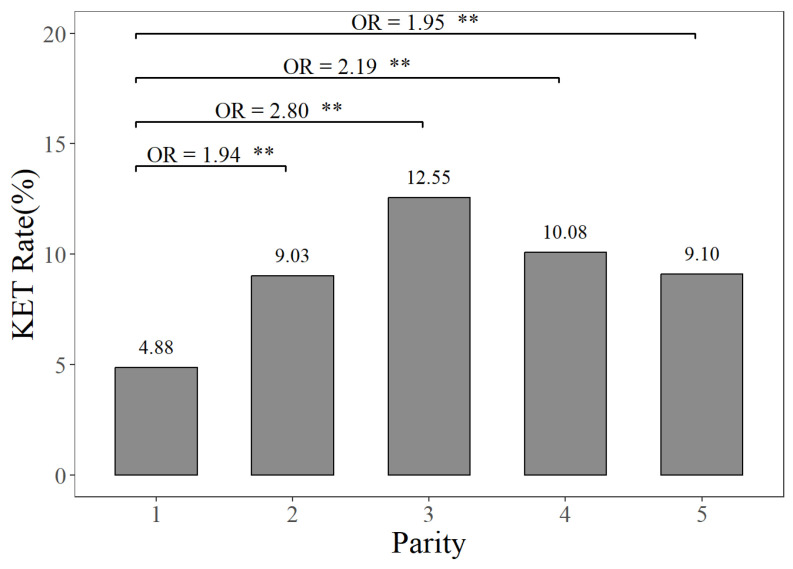
The prevalence rates and odds ratios (ORs) of ketosis in different parities, compared to the first parity. The KET Rate represents the ketosis prevalence rate. “**” represents no and highly significant differences (*p* < 0.01).

The prevalence rates of MAS, MET, and KET in different parities can be seen in Figure 1, Figure 2 and Figure 3. Figure 1 illustrates that the prevalence rate of MAS in the first parity was 16.30%, whereas the prevalence rates of MAS in the second to fifth parities ranged from 23.84% to 25.53%. The probabilities of MAS in the second, third, fourth, and fifth parities were 1.61, 1.75, 1.76, and 1.67 times higher, respectively, compared to the first parity (*p <* 0.01). It can be seen from Figure 2 that the prevalence rate of MET in the first parity was 10.20%, whereas the prevalence rates from the second parity to the fifth parities ranged from 9.10% to 11.88%. The probabilities of MET occurring in the second and third parities were 1.19 times and 1.13 times higher, respectively, compared to the prevalence rate of MET in the first parity (*p <* 0.01). According to Figure 3, the prevalence rates of KET were 4.88% in the first parity, and 9.03–12.55% in the second to fifth parities. The prevalence rates of KET in the second, third, fourth, and fifth parities were 1.94, 2.80, 2.19, and 1.95 times higher than that in the first parity, respectively (*p <* 0.01).

### 3.2. The Impacts of the Three Diseases on Milk Production, Milk Quality, and Reproductive Traits

**Figure 4:**
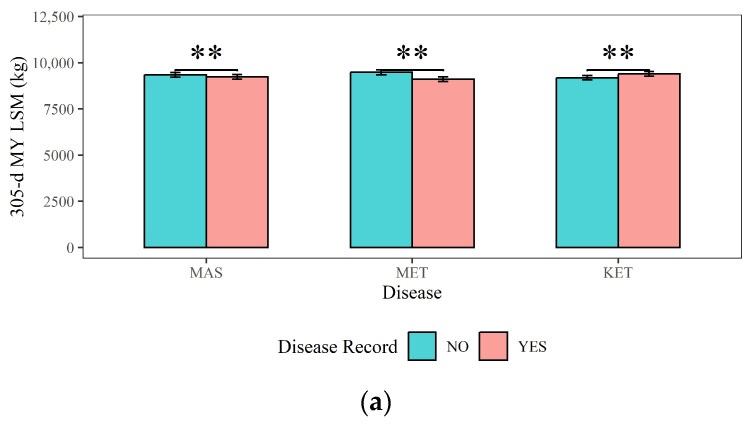

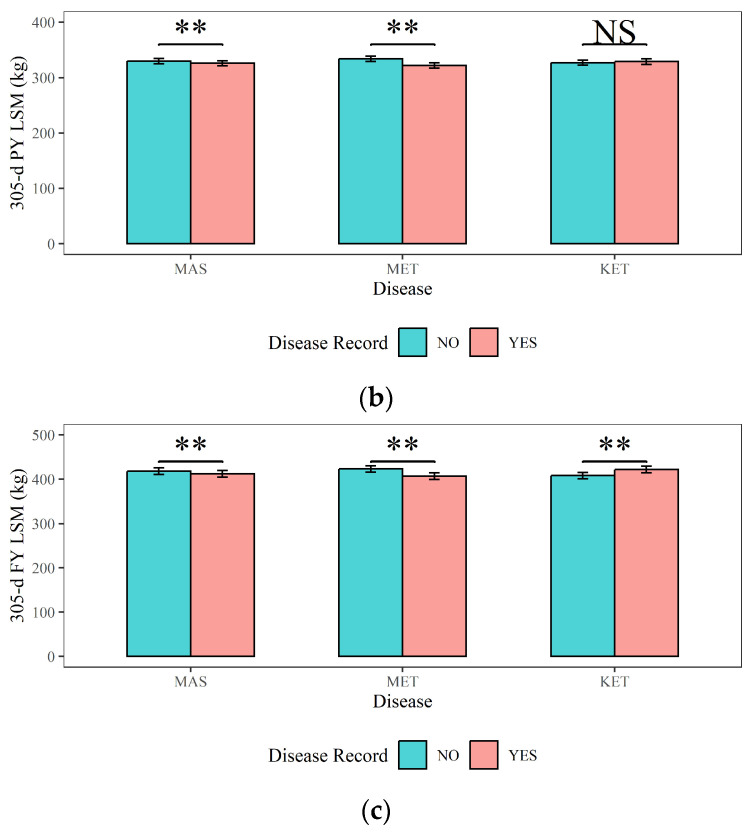
The effects of mastitis, endometritis, and ketosis on milk production in dairy cows. (**a**) 305-d MY represents 305-d Milk Yield, (**b**) 305-d PY represents 305-d Protein Yield, and (**c**) 305-d FY represents 305-d Fat Yield. MAS represents mastitis, MET represents endometritis, and KET represents ketosis. LSM represents the least squares means. NS and “**”, respectively, represent no and highly significant differences (*p >* 0.05, *p* < 0.01).

**Figure 5:**
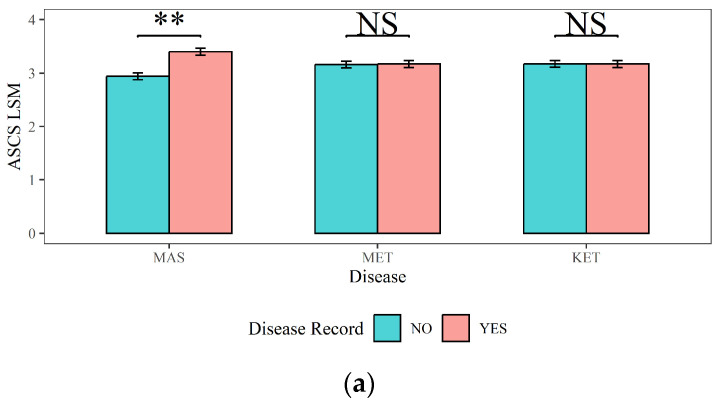

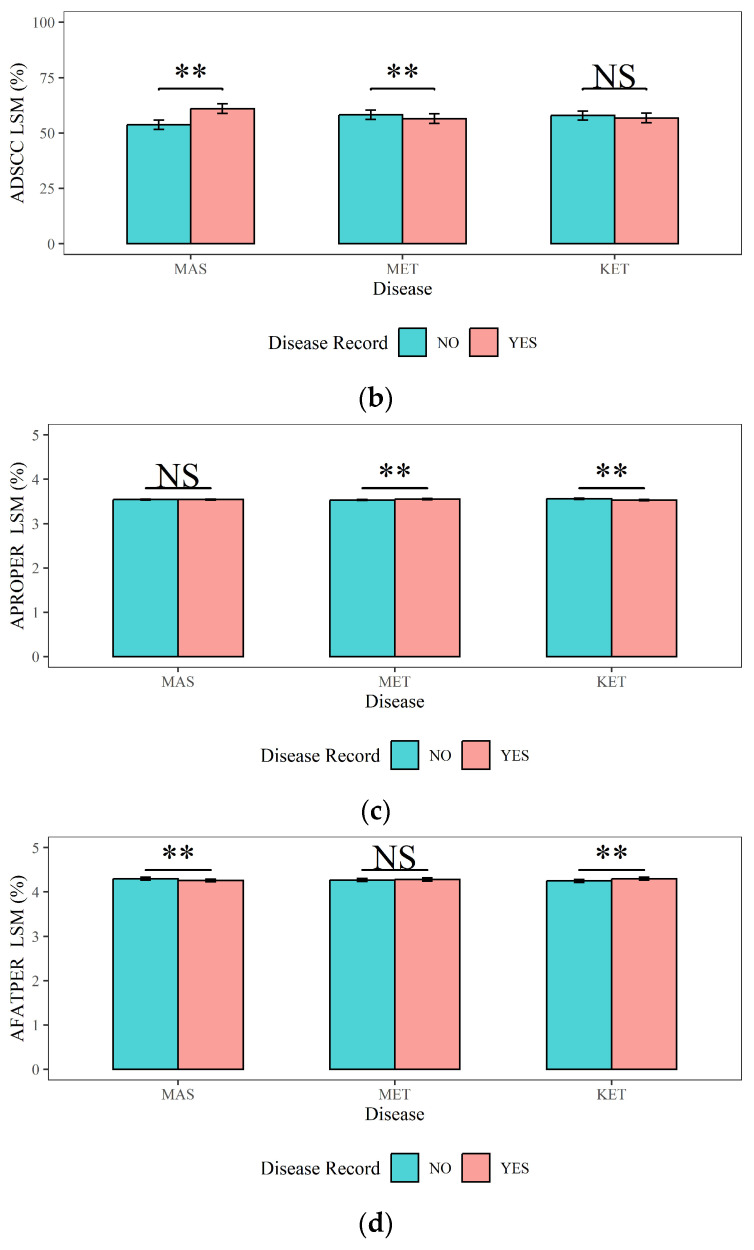
The effects of mastitis, endometritis, and ketosis on milk composition in dairy cows. (**a**) ASCS represents the Average Somatic Cell Score (SCS) in lactation, (**b**) ADSCC represents the Average Differential Somatic Cell Count (DSCC) in lactation, (**c**) APROPER represents the Average Protein Percentage in lactation, and (**d**) AFATPER represents the Average Fat Percentage in Lactation. MAS represents mastitis, MET represents endometritis, and KET represents ketosis. LSM represents the least squares means. NS and “**”, respectively, represent no and highly significant differences (*p >* 0.05, *p* < 0.01).

**Figure 6:**
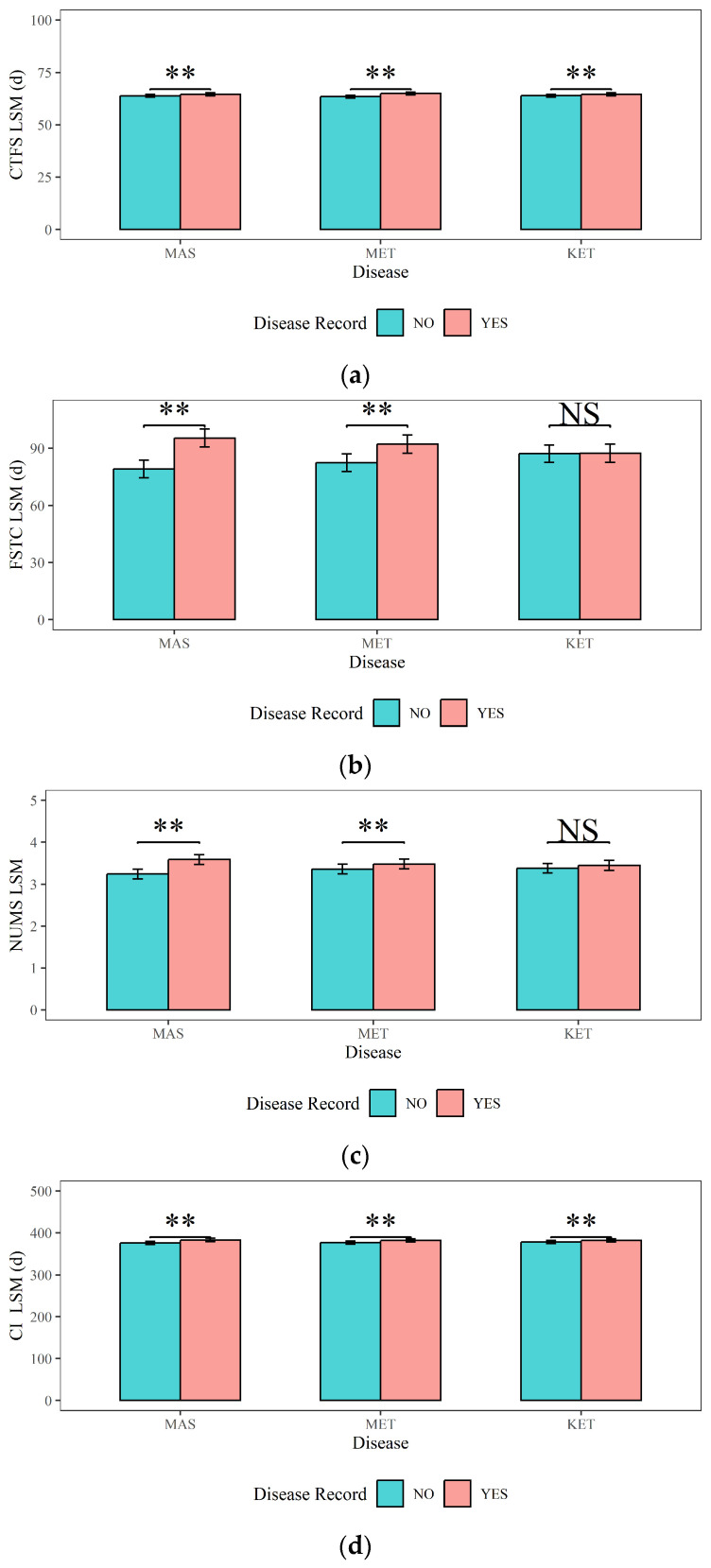
The effects of mastitis, endometritis, and ketosis on reproductive performance in dairy cows. (**a**) CTFS represents the Interval from Calving to First Service, (**b**) FSTC represents the Interval from First Service to Conception, (**c**) NUMS represents the Number of Services, and (**d**) CI represents Calving Interval. MAS represents mastitis, MET represents endometritis, and KET represents ketosis. LSM represents the least squares means. NS and “**”, respectively, represent no and highly significant differences (*p >* 0.05, *p* < 0.01).

Figure 4, Figure 5 and Figure 6 present a comprehensive overview of the effects of dairy cow MAS, MET, and KET on milk production, milk quality, and reproductive traits. Figure 4 demonstrates that (1) cows with MAS had considerably reduced 305-d MY, 305-d PY, and 305-d FY (9236 kg, 326 kg, and 412 kg) compared to cows without MAS (9348 kg, 330 kg, and 418 kg), with a statistical significance of *p <* 0.01; (2) The 305-d MY, 305-d PY, and 305-d FY (9106 kg, 322 kg, and 407 kg) of cows with MET were significantly (*p <* 0.01) lower than those of cows without MET (9478 kg, 334 kg, and 423 kg); (3) cows with KET had significantly higher 305-d MY and 305-FY (9395 kg and 422 kg) compared to cows without KET (9190 kg and 408 kg) (*p <* 0.01). This could be attributed to excessive milk production in the early lactation period, leading to a negative energy balance and the occurrence of KET.

The impacts of MAS, MET, and KET on the milk quality traits (ASCS, ADSCC, APROPER, and AFATPER) and their corresponding statistical significance were shown in Figure 5: (1) The ASCS and ADSCC in cows with MAS (3.40 and 61%) were significantly higher than those in cows without MAS (2.94 and 53.70%) (*p <* 0.01). The APROPER of dairy cows with MAS (4.26%) was lower than that of dairy cows without MAS (4.30%, *p <* 0.01). There was no significant effect of MAS on the APROPER (*p >* 0.05). (2) The ADSCC in cows with MET (56.50%) was significantly lower than that in cows without MET (58.20%) (*p <* 0.01). Cows with MET had a significantly higher APROPER (3.55%) compared to cows without MET (3.53%) (*p <* 0.01). However, MET did not have a significant influence on the ASCS or AFATPER (*p >* 0.05). (3) The APROPER of cows with KET (3.53%) was significantly lower than that of cows without KET (3.56%) (*p <* 0.01). The AFATPER of cows with KET (4.30%) was significantly higher (*p <* 0.01) than that of cows with no risk of KET (4.25%). KET had no significant influence on ASCS or ADSCC (*p >* 0.05).

Figure 6 illustrates the analyses results of the impacts of dairy cow MAS, MET, and KET on the reproductive traits (CTFS, FSTC, NUMS, and CI). Figure 6 shows that (1) the CTFS, FSTC, NUMS, and CI (64.50 d, 95.40 d, 3.59, and 383 d) of cows with MAS are highly significantly (*p <* 0.01) higher than those of cows without MAS (63.80 d, 79.20 d, 3.24, and 376 d). (2) Cows with MET had significantly higher CTFS (64.90 d), FSTC (92.20 d), NUMS (3.48), and CI (382 d) compared to cows without MET (63.40 d, 82.50 d, 3.36, and 377 d) (*p <* 0.01). (3) The CTFS and CI of cows with KET (64.50 d and 382 d) were significantly higher (*p <* 0.01) than those without KET (63.90 d and 378 d). However, there was no significant difference in the FSTC or NUMS in cows with KET (*p >* 0.05).

### 3.3. Genetic Analyses of the Three Diseases, Milk Production, Milk Quality, and Reproductive Traits

**Figure 7:**
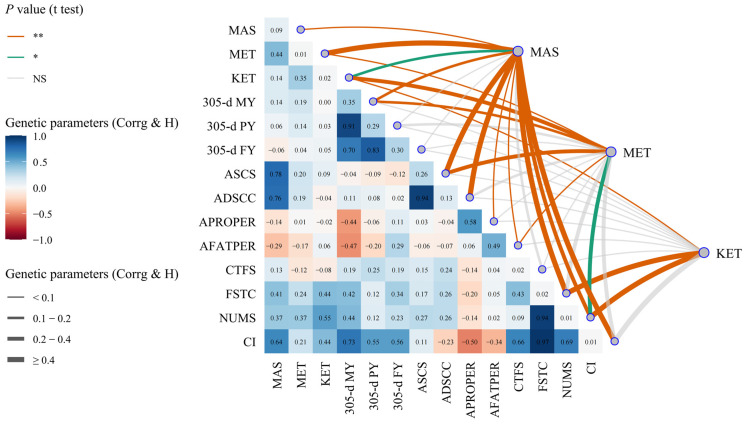
The heritability and genetic correlation of mastitis, endometritis, ketosis, milk yield, milk composition, and reproduction traits in dairy cattle. MAS represents mastitis, MET represents endometritis, KET represents ketosis, 305-d MY represents 305-d Milk Yield, 305-d PY represents 305-d Protein Yield, 305-d FY represents 305-d Fat Yield, ASCS represents the Average Somatic Cell Score (SCS) in lactation, ADSCC represents the Average Differential Somatic Cell Count (DSCC) in lactation, APROPER represents the Average Protein Percentage in lactation, AFATPER represents the Average Fat Percentage in lactation, CTFS represents the Interval from Calving to First Service, FSTC represents the Interval from First Service to Conception, NUMS represents the Number of Services, and CI represents the Calving Interval. Corrg, in the lower triangles, represents genetic correlation. H, in the diagonal, represents heritability. NS, “*”, and “**”, respectively, represent no, significant, and highly significant differences (*p >* 0.05, *p <* 0.05, *p* < 0.01).

**Figure 8:**
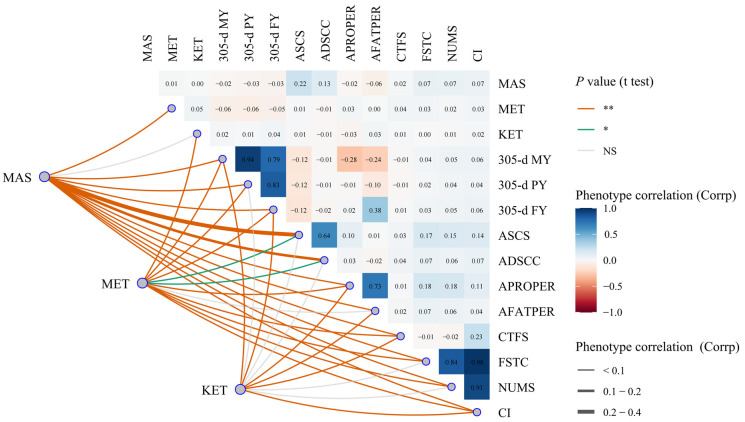
The phenotypic correlation of mastitis, endometritis, ketosis, milk yield, milk composition, and reproduction traits in dairy cattle. Corrp represents phenotypic correlation. NS, “*”, and “**”, respectively, represent no, significant, and highly significant differences (*p >* 0.05, *p <* 0.05, *p* < 0.01). The trait meanings are the same as in Figure 7.

The genetic parameters of dairy cow MAS, MET, and KET, milk production, milk quality, and reproductive traits are shown in Table 2 and Figure 7 and Figure 8. The data presented in Table 2 and Figure 7 indicate that (1) the heritabilities of cows with MAS, MET, and KET were 0.09, 0.01, and 0.02, respectively. Additionally, the genetic correlations between these diseases ranged from 0.14 to 0.44, all of which were statistically significant or highly significant (*p <* 0.05 or *p <* 0.01). (2) The heritabilities for milk production traits, namely 305-d MY, 305-d PY, and 305-d FY, ranged from 0.29 to 0.35. The genetic correlations between MAS or MET and 305-d MY were found to be 0.14 and 0.19, respectively (*p <* 0.01). However, the genetic correlations between MAS or MET and 305-d PY or 305-d FY were not found to be statistically significant (*p >* 0.05). Additionally, the genetic correlation between KET and milk production was also not found to be statistically significant (*p >* 0.05). (3) The heritabilities for milk quality traits, including ASCS and ADSCC, as well as APROPER and AFATPER, ranged from 0.13 to 0.58. The genetic correlations between MAS and the ASCS, ADSCC, APROPER, or AFATPER were 0.78, 0.76, −0.14, and −0.29, respectively (*p <* 0.01); the genetic correlations between MET and ASCS or AFATPER were highly significant, as 0.20 and −0.17, respectively (*p <* 0.01), while the genetic correlations between MET and ADSCC or APROPER were not significant (*p >* 0.05); the genetic correlations between KET and milk quality traits were between −0.04 and 0.09, not being significant (*p >* 0.05). (4) The heritabilities for reproductive traits, such as CTFS, FSTC, NUMS, and CI, ranged from 0.01 to 0.02; the genetic correlations between MAS and FSTC, NUMS, or CI were highly significant, ranging from 0.37 to 0.64 (*p <* 0.01); the genetic correlation between MAS and the CTFS was not statistically significant (*p >* 0.05); the genetic correlation between MET and NUMS was 0.37 (*p <* 0.05); the genetic correlations between MET and CTFS, FSTC or CI were not statistically significant (*p >* 0.05); the genetic correlations between KET and FSTC or NUMS were highly significant, with correlation coefficients of 0.44 and 0.55, respectively (*p <* 0.01); and the genetic correlations between KET and CTFS or CI were not statistically significant (*p >* 0.05).

The phenotypic correlations among traits are shown in Table 2 and Figure 8: (1) With the exception of KET, the phenotypic correlations between MAS and the other traits ranged from −0.06 to 0.22. All of these correlations were highly significant (*p <* 0.01). Specifically, the phenotypic correlation between MAS and ASCS was 0.22, and between MAS and ADSCC it was 0.13. However, there was no statistically significant correlation observed between MAS and KET (*p >* 0.05). (2) In addition to the APROPER, the phenotypic correlations between MET and the other traits ranged from −0.06 to 0.04, all of which were highly significant (*p <* 0.01). However, there was no significant phenotypic correlation between MET and APROPER (*p >* 0.05). (3) The phenotypic correlations between KET and 305-d MY, 305-d FY, APROPER, AFATPER, or CI ranged from −0.06 to −0.03, all of which were highly significant (*p <* 0.01). There was no significant phenotypic correlation between KET and the other traits (*p >* 0.05).

## 4. Discussion

Three diseases’ prevalence rates and their effects on milk production, milk quality, and reproductive traits: Studies reported that the prevalence rates of MAS and MET were 0.60–18.2% [1] and 3.4–53% [2,3], respectively. In this study, it was found that the overall prevalence rates of MAS and MET were 20.04% and 10.68%, respectively, which are similar to those reported in earlier studies. The prevalence rate of KET in this study (7.33%) was slightly lower than that observed in previous studies (10–30%) [4]. This discrepancy may be attributed to the dairy farm’s methodology for KET detection, which involved sampling only a subset of fresh cows within 100 days after calving, rather than all of them. The prevalence rate of MAS and KET tended to increase from the first to the fifth parities, whereas the prevalence rate of MET among parities ranged from 9.10 to 11.88%. The differences in the prevalence rate of these three diseases in dairy farms were related to the management methods. The fluctuations across different parities may be related to the increase in the age of cows and the decline in the resistance to disease.

The presence of MAS or MET showed negative effects on the 305-d MY, 305-d PY, and 305-d FY in this study. The 305-d MY, 305-d PY, and 305-d FY of cows with MAS or MET decreased by 112 kg and 372 kg (*p <* 0.01), 4 kg and 12 kg (*p <* 0.01), and 6 kg and 16 kg (*p <* 0.01), respectively, compared to cows without MAS or MET. These findings align with previous studies that have also reported milk loss due to MAS and MET [1,7]. The effects of KET on 305-d MY, 305-d PY, and 305-d FY were contrary to those of MAS or MET. When comparing cows with KET to cows without KET, it was shown that cows with KET had a significant increase in 305-d MY by 205 kg (*p <* 0.01), a non-significant increase in 305-d PY by 2 kg (*p >* 0.05), and a significant increase in 305-d FY by 14 kg (*p <* 0.01). In their study, Detilleux et al. discovered that although KET decreased the milk yield of dairy cows, the 305-d MY of cows with KET was greater (141.10 kg) compared to cows without KET [30]. Belay et al. demonstrated that dairy cows with KET during the early lactation stage (days in milk being lower than 80 days) exhibited increased daily milk yield and fat percentage compared to cows without KET. Additionally, these cows had a lower protein percentage, suggesting a greater probability of KET occurrence in high-yield cows [31]. We hypothesized that the incidence of KET was associated with the peak milk yield of dairy cows and the negative energy balance of fresh cows. The high-yield of the cows led to an elevation in the ketone body content in their bodies, owing to disruptions in glucose metabolism.

The study found that MAS, MET, and KET had negative impacts on four reproductive traits: CTFS, FSTC, NUMS, and CI. The negative impacts may be attributed to abnormal hormone secretion [32], delayed resumption of ovarian activity [3], and slowed cluster duration and activity times during the estrus stage [33]. Dairy cows with MAS, MET, or KET experienced an increase in various reproductive traits compared to cows without disease records. Specifically, CTFS increased by 0.60–1.5 days, FSTC increased by 0.20–16.20 days, NUMS increased by 0.07–0.35, and CI increased by 4–7 days. With the exception of the effect of KET on FSTC or NUMS, all other factors were highly significant (*p <* 0.01). This is comparable to other research reports [2,6,7,33].

The genetic parameters of the three diseases in dairy cows: This study gathered the health diagnostic records of the dairy farm to examine the prevalence rates of MAS, MET, and KET, their effects on other traits, and to do genetic analyses. Laboratory pathogen isolation and testing, cytological detection methods, and high-performance liquid chromatography for ketone in the blood are the “gold standards” for the detection of dairy cow MAS, MET, and KET. However, these technologies often exhibit drawbacks such as exorbitant detection expenses, lengthy detection duration, stringent sample criteria, and an inability to perform extensive-scale detection. Consequently, they provide a limited quantity of illness phenotypic data, hence impeding the genetic study of diseases [34]. Previous research’s findings suggested a strong genetic correlation (0.98–0.99) [35] between diseases recorded in the health diagnosis records of dairy farms and the laboratory detection records of dairy cow MAS, retained placenta, and milk fever. This indicates that the health diagnosis records data from dairy farms can be effectively utilized for genetic analyses of these diseases [18,19].

The heritability of cows with MAS, MET, or KET in this study was found to be 0.09, 0.01, and 0.02, respectively. These values indicated that these diseases had low heredity, which aligns with the findings of previous studies where the heritability of cows with MAS ranged from 0.01 to 0.12, MET ranged from 0.02 to 0.03, and KET ranged from 0.03 to 0.16 [11,16,17,18,19]. The genetic correlations of dairy cow MAS, MET, and KET ranged from 0.14 to 0.44 (*p <* 0.01). Specifically, the genetic connection between MAS and KET was 0.14. Heringstad B et al. conducted a study that revealed the genetic correlation between MAS and KET of 1 to 3 parities from 0.16 to 0.26 (*p <* 0.01) [36]. Hardie et al. demonstrated that the genetic correlation between MAS and MET was 0.06 (*p >* 0.05), whereas the genetic correlation between MAS and KET was 0.19 (*p <* 0.05) [19]. Therefore, the breeding strategies used to control one of the diseases may impact the incidence of the other two diseases. The genetic correlation between MAS and various traits, such as 305-d MY, ASCS, ADSCC, FSTC, NUMS, or CI, was found to be between 0.14 and 0.78 (*p <* 0.01). The highest genetic correlation was observed with the ASCS. Additionally, the genetic correlations with APROPER and AFATPER were −0.14 and −0.29 (*p <* 0.01), respectively. The genetic connections between MET and 305-d MY, ASCS, or NUMS were 0.19, 0.20, and 0.37, respectively (*p <* 0.01 or *p <* 0.05). Additionally, the genetic correlation between MET and AFATPER was −0.17 (*p <* 0.01). The genetic correlations between KET and FSTC or NUMS were shown to be moderate (*p <* 0.01).

## 5. Conclusions

This study comprehensively investigated the prevalence rates of MAS, MET, and KET in Central China. It also examined the consequences of these diseases on critical attributes of dairy cows and performed a genetic analysis. The primary findings encompass the following: (1) The prevalence rates of MAS, MET, and KET in dairy cows in Central China were 20.04%, 10.68%, and 7.33%, respectively. Furthermore, the prevalence rates of MAS and KET increased progressively from the first parity to the fifth parity, while the prevalence rates of MET ranged between 9.10% and 11.88% across different parities. (2) The three diseases had distinct impacts on milk quality, particularly on the traits of APROPER and AFATPER. MAS had a highly significant effect on the AFATPER, while MET had a significant effect on the APROPER. KET had significant effects on both APROPER and AFATPER (*p <* 0.01). Additionally, both MAS and MET resulted in a decrease in 305-d MY, as well as in 305-d PY and 305-d FY. The 305-d MY of dairy cows with KET was significantly more substantial than that of non-ketotic dairy cows (205 kg, *p <* 0.01), maybe resulting from the excessive milk production that disrupts sugar metabolism in ill dairy cows. (3) The three diseases increased the number of days between CTFS, FSTC, CI, and NUMS. The effects of MAS or MET in dairy cows on FSTC or NUMS were highly significant (*p <* 0.01). (4) MAS, MET, and KET were low-heritable traits, with a genetic correlation ranging from 0.14 to 0.44 among the three diseases. The findings of this study provide information for disease management and the development of disease-resistant breeding strategies.

## Figures and Tables

**Table 1:** Detailed information of traits.

Type	Traits	Abbreviations	Unit	Meaning
Disease				
	Mastitis	MAS	/	The presence of illness records during the cow’s lactation period, a binary trait, where 0 indicates no illness records and 1 indicates the presence of illness records.
	Endometritis	MET	/	
	Ketosis	KET	/	
Milk quality				
	Average Somatic Cell Score (SCS) in Lactation	ASCS	/	/
	Average Differential Somatic Cell Count (DSCC) in Lactation	ADSCC	%	/
	Average Protein Percentage in Lactation	APROPER	%	/
	Average Fat Percentage in Lactation	AFATPER	%	/
Milk production				
	305-d Milk Yield	305-d MY	kg	305-d milk yield during the lactation period.
	305-d Protein Yield	305-d PY	kg	305-d milk protein yield during the lactation period.
	305-d Fat Yield	305-d FY	kg	305-d milk fat yield during the lactation period.
Reproduction				
	Interval from Calving to First Service	CTFS	d	The interval days between the calving date and the first service date.
	Interval from First Service to Conception	FSTC	d	The interval days between the first service date and conception date.
	Number of Services	NUMS	/	/
	Calving Interval	CI	d	The interval days between the calving dates.

**Table 2:** Heritability, phenotypic, and genetic correlations of cows with mastitis, endometritis, ketosis, milk yield, milk composition, and reproduction traits.

Traits	MAS	MET	KET	305-d MY	305-d PY	305-d FY	ASCS	ADSCC	APROPER	AFATPER	CTFS	FSTC	NUMS	CI
MAS	0.09 (0.00)	0.01 (0.00)	0.00 (0.00)	−0.02 (0.01)	−0.03 (0.01)	−0.03 (0.01)	0.22 (0.00)	0.13 (0.01)	−0.02 (0.00)	−0.06 (0.00)	0.02 (0.00)	0.07 (0.00)	0.07 (0.00)	0.07 (0.01)
MET	0.44 (0.08)	0.01 (0.00)	0.05 (0.00)	−0.06 (0.01)	−0.06 (0.01)	−0.05 (0.01)	0.01 (0.00)	−0.01 (0.01)	0.03 (0.00)	0.00 (0.00)	0.04 (0.00)	0.03 (0.00)	0.02 (0.00)	0.03 (0.01)
KET	0.14 (0.06)	0.35 (0.11)	0.02 (0.00)	0.02 (0.01)	0.01 (0.01)	0.04 (0.01)	0.01 (0.00)	−0.01 (0.01)	−0.03 (0.00)	0.03 (0.00)	0.01 (0.00)	0.00 (0.00)	0.01 (0.00)	0.02 (0.01)
305-d MY	0.14 (0.03)	0.19 (0.07)	0.00 (0.05)	0.35 (0.01)	0.94 (0.00)	0.79 (0.01)	−0.12 (0.01)	−0.01 (0.01)	−0.28 (0.01)	−0.24 (0.01)	−0.01 (0.01)	0.04 (0.01)	0.05 (0.01)	0.06 (0.01)
305-d PY	0.06 (0.04)	0.14 (0.08)	0.03 (0.07)	0.91 (0.00)	0.29 (0.01)	0.83 (0.01)	−0.12 (0.01)	−0.01 (0.01)	−0.01 (0.01)	−0.10 (0.01)	−0.01 (0.01)	0.02 (0.01)	0.04 (0.01)	0.04 (0.01)
305-d FY	−0.06 (0.04)	0.04 (0.08)	0.05 (0.07)	0.70 (0.01)	0.83 (0.01)	0.30 (0.02)	−0.12 (0.01)	−0.02 (0.01)	0.02 (0.01)	0.38 (0.01)	0.01 (0.01)	0.03 (0.01)	0.05 (0.01)	0.06 (0.02)
ASCS	0.78 (0.02)	0.20 (0.07)	0.09 (0.05)	−0.04 (0.02)	−0.09 (0.03)	−0.12 (0.03)	0.26 (0.01)	0.64 (0.01)	0.10 (0.00)	0.01 (0.00)	0.03 (0.01)	0.17 (0.01)	0.15 (0.01)	0.14 (0.01)
ADSCC	0.76 (0.04)	0.19 (0.10)	−0.04 (0.09)	0.11 (0.04)	0.08 (0.05)	0.02 (0.05)	0.94 (0.01)	0.13 (0.01)	0.03 (0.01)	−0.02 (0.01)	0.04 (0.01)	0.07 (0.01)	0.06 (0.01)	0.07 (0.01)
APROPER	−0.14 (0.02)	0.01 (0.05)	−0.02 (0.04)	−0.44 (0.02)	−0.06 (0.02)	0.11 (0.02)	0.03 (0.02)	−0.04 (0.03)	0.58 (0.01)	0.73 (0.00)	0.01 (0.01)	0.18 (0.01)	0.18 (0.01)	0.11 (0.01)
AFATPER	−0.29 (0.02)	−0.17 (0.06)	0.06 (0.04)	−0.47 (0.02)	−0.20 (0.02)	0.29 (0.02)	−0.06 (0.02)	−0.07 (0.03)	0.06 (0.00)	0.49 (0.01)	0.02 (0.01)	0.07 (0.01)	0.06 (0.01)	0.04 (0.01)
CTFS	0.13 (0.09)	−0.12 (0.14)	−0.08 (0.12)	0.19 (0.08)	0.25 (0.09)	0.19 (0.09)	0.15 (0.07)	0.24 (0.12)	−0.14 (0.06)	0.04 (0.06)	0.02 (0.00)	−0.01 (0.00)	−0.02 (0.00)	0.23 (0.01)
FSTC	0.41 (0.08)	0.24 (0.13)	0.44 (0.10)	0.42 (0.07)	0.12 (0.09)	0.34 (0.09)	0.17 (0.07)	0.26 (0.12)	−0.20 (0.06)	0.05 (0.06)	0.43 (0.12)	0.02 (0.00)	0.84 (0.00)	0.98 (0.00)
NUMS	0.37 (0.10)	0.37 (0.15)	0.55 (0.11)	0.45 (0.09)	0.12 (0.10)	0.23 (0.10)	0.27 (0.09)	0.26 (0.14)	−0.14 (0.07)	0.02 (0.07)	0.09 (0.17)	0.94 (0.02)	0.01 (0.00)	0.91 (0.00)
CI	0.64 (0.13)	0.21 (0.26)	0.44 (0.22)	0.73 (0.11)	0.55 (0.15)	0.56 (0.14)	0.11 (0.14)	−0.23 (0.22)	−0.50 (0.13)	−0.34 (0.14)	0.66 (0.18)	0.97 (0.01)	0.69 (0.10)	0.01 (0.00)

Note: The diagonal represents heritability and its standard error, while the upper and lower triangles represent phenotypic correlation, additive correlation, and their corresponding standard errors. MAS represents mastitis, MET represents endometritis, KET represents ketosis, 305-d MY represents 305-d Milk Yield, 305-d PY represents 305-d Protein Yield, 305-d FY represents 305-d Fat Yield, ASCS represents the Average Somatic Cell Score (SCS) in lactation, ADSCC represents the Average Differential Somatic Cell Count (DSCC) in lactation, APROPER represents the Average Protein Percentage in lactation, AFATPER represents the Average Fat Percentage in lactation, CTFS represents the Interval from Calving to First Service, FSTC represents the Interval from First Service to Conception, NUMS represents the Number of Services, and CI represents the Calving Interval.

## Data Availability

The data presented in this study are available on request from the corresponding author due to data ownership reasons.
